# The Influence of the Ball Milling Process on the Structure and Functional Properties of Walnut Meal

**DOI:** 10.3390/foods15132250

**Published:** 2026-06-23

**Authors:** Yanyue Li, Yanling Lu, Yanmei Deng, Lei Guo, Long Han, Qian Ma, Fangyu Fan

**Affiliations:** 1 College of Biological and Food Engineering, Southwest Forestry University, Kunming 650224, China; liyanyue1217@163.com (Y.L.); luyanling20220222@163.com (Y.L.); 14736649465@163.com (Y.D.); guoleigift.student@sina.com (L.G.); longhan24yn@163.com (L.H.); maqian0224@163.com (Q.M.); 2 Key Laboratory of Forest Disaster Warning and Control of Yunnan Province, Kunming 650224, China; 3 Key Laboratory of National Forestry and Grassland Administration on Biodiversity Conservation in Southwest China, Southwest Forestry University, Kunming 650224, China

**Keywords:** walnut residue, mechanical grinding, protein and insoluble dietary fiber, structure and functional properties

## Abstract

To evaluate the potential of defatted and dephenolized walnut meal as a modified functional food ingredient, this study examined how ball milling and processing time affect its structural, physicochemical, and functional properties. Walnut meal was ball-milled for 5, 10, 15, and 20 h. Ball milling increased the lightness and whiteness, reduced particle size, and broadened the particle size distribution into a characteristic three-peak pattern. Scanning electron microscopy revealed the progressive formation of flake-like surface structures. With increasing milling duration, free sulfhydryl groups, surface hydrophobicity, and solubility were increased, while dynamic surface tension decreased, leading to improved foaming capacity and foaming stability. SDS-PAGE confirmed that the primary structure remained unchanged, while Fourier transform infrared spectroscopy indicated a decrease in α-helix and β-sheet contents and an increase in random coil structures. X-ray diffraction revealed a reduction in the diffraction peak at 2θ = 8.963°, and differential scanning calorimetry showed irregular changes in the thermal stability with ball milling time. Overall, increasing ball milling time is beneficial for improving the functional properties of walnut meal, providing a preliminary theoretical reference for the potential application of walnut powder in foods with specific functional properties, such as aerated foods.

## 1. Introduction

Over time, people’s interest in popular dietary trends has increased, leading to varied changes in dietary habits. There is a growing demand for food ingredients that are naturally derived, healthy, and sustainably sourced [1]. Walnut meal is an inexpensive, underutilized, and nutrient-rich byproduct of walnut processing [2]. Dephenolization and defatting of walnut meal not only preserve the key nutrients (protein and insoluble dietary fiber, IDF) but also enhance its storage stability. Its protein components mainly consist of glutelins, globulins, albumins, and prolamins [3,4]. Walnut protein is particularly abundant in arginine, glutamic acid, histidine, and tyrosine [5]. Another important component of walnut meal is dietary fiber (DF), which may effectively promote the growth of gut microbiota, exert prebiotic effects, and help prevent intestinal diseases such as colon cancer [6,7,8].

The utilization of walnut protein in food is lower than that of milk and egg white proteins because of its solubility, which is typically below 30%, and its high glutelin and prolamin [9]. In walnut meal, the molecular architecture of IDF favors extensive intermolecular hydrogen bonding and high crystallinity, forming fibrillar structures that exhibit high stiffness and nonlinear mechanical behavior. Humans lack the enzymatic capacity to hydrolyze β(1,4)-glycosidic bonds, making IDF difficult to digest and utilize efficiently [10]. Therefore, rather than being fully utilized, walnut meal is often fed to livestock or discarded, wasting abundant nutritional resources [3,11].

Ultrafine grinding, particularly ball milling (BM), is widely employed to enhance food ingredient functionality due to its low cost [12]. Studies have shown that ball milling can enhance the structural and functional properties of DF and proteins [13,14]. Ball milling has been applied to extract nanocellulose from walnut shells, tailor walnut meal for paste production, and enhance the structural and functional properties of walnut meal proteins [15,16]. However, the technology has not been widely applied to whole walnut meal. In contrast to these established applications, little information is available on the structural and functional properties of ball-milled whole walnut meal.

In summary, this study aimed to assess the feasibility of modifying walnut meal using ball milling and to characterize the milling duration-dependent changes, particularly in structural and functional properties. Correlation analysis was employed to explore the relationships between structural changes and functional properties. The research findings provide a theoretical basis for the direct application of the ball milling method to modify walnut meal.

## 2. Materials and Experimental Protocol

### 2.1. Materials

Fresh walnuts were harvested from Yangbi County (Dali, Yunnan, China) and selected as the raw experimental material after manual screening to remove deteriorated and immature fruits. The walnut meal utilized in this study was self-prepared in our laboratory as a major byproduct during walnut oil extraction. Briefly, walnut kernels were pressed under standardized processing conditions to remove crude oil, and the residual defatted walnut meal was collected, crushed, sieved, and fully dried for subsequent experimental analysis. All chemical reagents applied in the present work were of analytical grade and used without further purification.

### 2.2. Preparation of Walnut Meal

Fresh walnuts were air-dried naturally and then subjected to oil extraction using a screw press at 140 °C, producing walnut meal as a byproduct. The walnut meal was sequentially defatted with petroleum ether and dephenolized with 75% ethanol; each solvent extraction was performed three times with stirring at 100 rpm for 45 min. After drying at 40 °C for 12 h, the walnut meal was passed through a 60-mesh sieve and stored at room temperature in a dry environment.

### 2.3. Ball Milling Procedure

**Figure 1:**
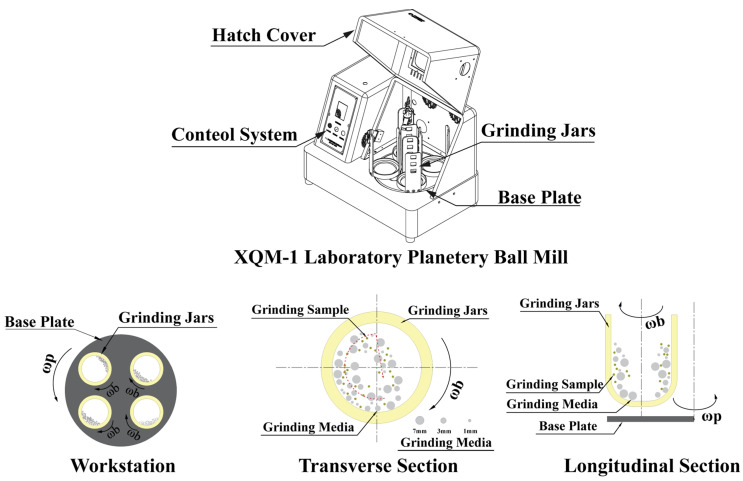
Schematic representation of the ball milling principle.

The operation is shown in Figure 1. Grinding beads with diameters of 1, 3, and 7 mm were mixed with walnut meal at a 1:1 (*w*/*w*) ratio. The sample was placed into the milling jar (XQM-1, Tianchuang Powder Technology Co., Ltd., Changsha, China), and two jars were mounted symmetrically to balance the mill. The mill was operated at 600 rpm, reversing direction and resting for 1 min after every 5 min. Walnut meal samples with different ball milling times were designated as BM-0, BM-5, BM-10, BM-15, and BM-20, respectively [17,18].

### 2.4. Proximate Composition Analysis of Walnut Meal

The moisture content of walnut meal was determined by oven-drying at 105 °C to a constant weight, as described by Jiang et al. [19]. Protein, DF, and ash contents were determined in accordance with the National Food Safety Standards of China: GB 5009.5-2025 [20], GB 5009.88-2023 [21], and GB 5009.4-2016 [22], respectively.

### 2.5. Determination of Color

Color was analyzed using a spectrophotometer (CS-5960GX, Hangzhou Caipu Technology Co., Ltd., Hangzhou, China). L⁎, a⁎, and b⁎ represent the lightness, redness-greenness, and yellowness-blueness indices, respectively. Whiteness (W) and color difference (ΔE) were calculated as follows:

(1) $$W=100-\sqrt{(100-L*)+a{*}^{2}+b{*}^{2}}$$

(2) $$\Delta E=\sqrt{{\left(L*-L_{0}\right)}^{2}+{\left(a*-a_{0}\right)}^{2}+{\left(b*-b_{0}\right)}^{2}}$$

L_0_, a_0_, and b_0_ denote the control values of the reference standard, whereas L⁎, a⁎, and b⁎ represent the color parameters of samples processed at different ball milling times.

### 2.6. Particle Size and Zeta Potential Analysis

The particle size and zeta potential were determined using the Mastersizer 2000 and Zs 90 (Malvern Panalytical Ltd., Worcestershire UK), respectively. Dispersion was performed in distilled water with a refractive index of 1.33. The mean particle diameter is represented by the volume-weighted D_[4,3]_ and the surface-weighted D_[3,2]_.

### 2.7. SEM Analysis

Samples were evenly spread onto conductive carbon tape using a cotton swab and sputter-coated with gold for 30 s. SEM imaging was performed on a Sigma 360 (Carl ZEISS Vision International GmbH, Aalen, Germany). Images were acquired at magnifications of 1000× and 2000× to observe changes in the surface morphology of ball-milled walnut meal.

### 2.8. SDS-PAGE Analysis

SDS-PAGE was conducted according to Ma et al. [23], with minor modifications. Gel plates were prepared using 5% stacking gel and 15% separating gel. Samples were dissolved in a phosphate-buffered saline (PBS, pH 7.0, 10 mM) at 1.0 mg/mL and mixed with the sample loading buffer at a 4:1 (*v*/*v*) ratio. The mixture was subjected to a boiling water bath for 10 min, followed by cooling. Sample solutions (5 µL) from different ball milling times and protein molecular weight marker (5 μL) were loaded into the wells. Electrophoresis was performed at 80 V for 1 h, followed by 120 V for 2 h. Gels were stained with Coomassie Brilliant Blue and destained with a methanol-glacial acetic acid solution for 24 h.

### 2.9. FTIR Analysis

FTIR measurements were performed following Kobayashi et al. [24], with minor modifications. The sample was mixed and ground with potassium bromide at a mass ratio of 1:100 (*w*/*w*). The mixture was then pressed for 30 s. The infrared spectra were obtained using a spectrometer (FTIR-650, Gangdong Technology Co., Ltd., Tianjin, China) over a range of 400–4000 cm^−1^ with 32 scans at a resolution of 4 cm^−1^. Finally, the data were processed with OMNIC v8.2, followed by deconvolution using Peakfit v4.12 for protein structure analysis.

### 2.10. XRD Analysis

XRD patterns were obtained by an X-ray diffractometer (Ultima IV, Rigaku, Tokyo, Japan). The measurements followed Ahmad et al. [25], with slight modifications. XRD patterns were recorded using Cu-Kα radiation (λ = 1.5406 Å) over a scan range of 5–50° (2θ) at a scan rate of 5°/min. The relative crystallinity was calculated by Gaussian deconvolution of crystalline peaks and the amorphous background.

### 2.11. DSC Analysis

Experiments were conducted to determine the thermal stability of the samples using a DSC-204 F1 instrument (NETZSCH, Selb, Germany), according to Li et al. [26], with slight modifications. The temperature program was 25–200 °C at a heating rate of 10 °C/min under a nitrogen atmosphere (50 mL/min).

### 2.12. Free Sulfhydryl (-SH) Analysis

The -SH content was determined following Deng et al. [27], with minor modifications. Walnut meal was suspended in Tris-glycine buffer at a concentration of 5 mg/mL, followed by the addition of 200 μL of DTNB solution. The mixture was incubated at room temperature for 20 min. After centrifugation at 4000× *g* for 15 min, the supernatant was collected. The absorbance of the sample was measured at 412 nm using a UV-VIS spectrophotometer (UV-2700, Shimadzu, Kyoto, Japan). The disulfide bond content is calculated as half of the difference between total sulfhydryl content and free sulfhydryl content. The -SH content was calculated using Equation (3):

(3) $$\text{-}\mathrm{SH}(\mu \mathrm{mol}/g)=\frac{73.53\times A_{412}\times D}{C}$$

where A_412_ is the absorbance of the sample at 412 nm; D represents the dilution factor; C is the mass concentration of the sample (mg/mL); and 73.53 is the molar extinction coefficient of Ellman’s reagent.

### 2.13. Surface Hydrophobicity (H_0_) Analysis

According to Deng et al. [28], the H_0_ of walnut meal was determined using ANS reagent as a fluorescent probe, with minor modifications. Walnut meal in PBS (10 mM, pH 7.2) was centrifuged (4200× *g*, 5 min). The supernatant was used to prepare 0.2, 0.4, 0.6, 0.8, and 2.0 mg/mL solutions, which were mixed with 50 μL of 8 mM ANS (8-anilino-1-naphthalenesulfonic acid) solution and incubated in the dark at 25 °C for 30 min. Using a fluorescence spectrophotometer (F-2710, Hitachi, Tokyo, Japan) with an excitation/emission wavenumber of 390/470 nm and a slit width of 5 nm, the H_0_ value was determined as the slope of the linear regression of fluorescence intensity against protein concentration.

### 2.14. Solubility Analysis

Protein solubilization was evaluated based on Huang et al. [29], with minor adjustments. A 100 mg sample was dissolved in distilled water to prepare a 1 mg/mL solution. The solution was vortexed and then centrifuged at 8000× *g* for 5 min. The nitrogen content in the initial solution (before centrifugation) and in the supernatant (after centrifugation) was determined separately by the Kjeldahl method. Protein solubilization capacity (%) was calculated using Equation (4).

(4) $$\mathrm{solubility}\;(\%)=\frac{m_{0}}{m_{1}}\times 100$$

with m_0_ and m_1_ being the protein concentrations in the supernatant and the sample, respectively.

### 2.15. Foaming Capacity (FC) and Foam Stability (FS) Analysis

FC and FS were determined according to Liu et al. [30], with slight modifications. A 100 mL sample suspension (1 mg/mL) was prepared in a graduated cylinder and homogenized at 10,000 rpm for 1 min. Calculations were performed using Equations (5) and (6).

(5) $$\mathrm{FC}\;(\%)=\frac{V_{1}-V}{V}\times 100$$

(6) $$\mathrm{FS}(\%)=\frac{V_{2}-V}{V_{1}-V}\times 100$$

where V is the initial liquid volume (100 mL in this study), V_1_ is the total volume of solution and foam immediately after homogenization, and V_2_ is that after standing for 20 min.

### 2.16. Optical Microscopy Analysis

Walnut meal (100 mg) was placed in a 300 mL graduated cylinder, and then 100 mL of distilled water was added to prepare a 0.1% (*w*/*v*) walnut meal suspension. The mixture was vortexed for 1 min and then subjected to high-speed dispersion at 12,000× *g* for 2 min. The resulting dispersion was allowed to stand for 30 min, and an appropriate amount of foam was transferred onto a glass slide. To avoid bubble rupture, no coverslip was applied. The morphology of the walnut meal foam was examined at 10× magnification.

### 2.17. Dynamic Surface Tension Analysis

Determination of dynamic surface tension followed the procedure described by Maurya et al. [31], with minor modifications. A 1% (*w*/*v*) sample solution was prepared in PBS (10 mM, pH 7.0). The dynamic surface tension was measured using a DCAT21 tensiometer (SITA, Dresden, Germany), typically employing the Wilhelmy plate method.

### 2.18. Data Processing and Statistical Analysis

All experiments were performed in triplicate at a minimum, and initial data processing was conducted by Microsoft Excel 2021. Statistical analysis was conducted using IBM SPSS, version 27.0 (IBM Crop., Armonk, NY, USA). One-way analysis of variance (ANOVA), followed by Duncan’s multiple range test, was applied, and the significance level was set at *p* < 0.05. Figures were generated in Origin 2021. Results are expressed as mean ± SD.

## 3. Results and Discussion

### 3.1. Component Contents of Walnut Meal

According to Table 1, the major components of walnut meal were as follows: protein had the highest content (68.85%), followed by ash (7.15%). The contents of SDF and IDF were 5.30% and 11.77%, respectively, whereas the moisture content was the lowest at 4.89%.

### 3.2. Color Analysis

Color is a key sensory attribute of milled products and plays an important role in food production and processing. Therefore, color analysis was conducted on walnut meal subjected to different milling times, and Table 2 presents the color parameters corresponding to these different milling times. After ball milling, the L⁎, b⁎, and W values of walnut meal changed significantly (*p* < 0.05). The increase in the W value may be attributed to mechanical impacts (e.g., collisions and friction) that disrupt particle integrity, expose fresher inner surfaces, and reduce particle size, thereby enhancing light scattering and increasing whiteness. Consistently, L⁎ value increased, likely due to the same factors (finer particles and fresher surfaces), which enhance diffuse reflectance. The b⁎ value indicates the yellowness of samples. Further analysis suggests that the typical yellowness of walnut meal is likely related to Maillard reactions occurring during upstream processing. The augmentation of b⁎ may be attributed to particle size reduction, which increases the specific surface area and accelerates oxidative reactions, thereby slightly intensifying yellow chromophores. Ball milling altered walnut meal color parameters (increased L⁎, W, and b⁎), thereby enhancing perceived whiteness and product appearance [32].

### 3.3. Particle Size Distribution and Zeta Potential

**Figure 2:**
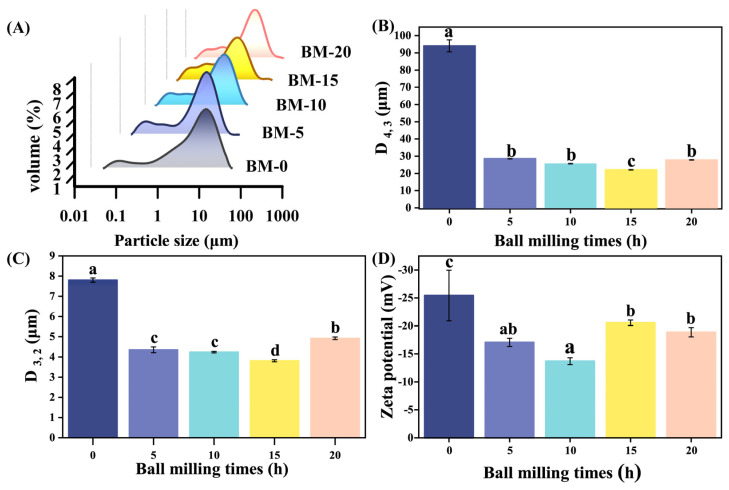
Particle size distribution (**A**), D_[4,3]_ (**B**), D_[3,2]_ (**C**), and zeta potential (**D**) of walnut meal at different ball milling times. Different letters indicate significant differences between different milling durations (*p* < 0.05) by Duncan’s test.

The effect of ball milling duration on walnut meal particle size distribution is illustrated in Figure 2A. BM-0 showed a bimodal particle size distribution, with a sharp peak in the 50–200 μm range, accounting for 55.43% by volume. This indicates severe aggregation of the original particles with a concentrated size distribution. After 15 h of ball milling, BM-15 exhibited a clear shift in the main peak to the range of 10–50 μm, with a volume fraction of 49.49%, accompanied by peak broadening. Additionally, a distinct peak appeared at 1–10 μm with a volume fraction of 32.17%, forming a typical three-peak morphology. This indicated that the mechanical energy provided by ball milling effectively destroyed aggregates, refined the particles, and led to a multi-level size distribution. Meanwhile, the broadening of the peak shape reflected an increase in particle size diversity, which was beneficial for improving particle packing efficiency and surface contact area. These results were similar to those previously reported by Zhu et al. [33]. However, BM-20 exhibited a volume fraction of 22.76% in the 1–10 μm range, while the overall distribution shifted toward the 10–50 μm range, accounting for a volume fraction of 55.67%, which was an increase of 6.81% compared to BM-15’s volume fraction in the same range. The peak shape in the 10–50 μm interval became sharper, whereas the peak in the 1–10 μm interval became lower. This phenomenon indicated that over-grinding induces article agglomeration, with a sharp increase in newly formed micron-sized particles. These particles underwent secondary agglomeration via van der Waals forces, forming larger agglomerates. In Figure 2B,C, BM-15 exhibited the smallest mean particle size and specific surface area. In contrast, for BM-20, the mean particle size increased from 22.10 μm to 27.86 μm, and the specific surface area increased from 3.78 m^2^/g to 4.96 m^2^/g. The formation of micron-sized particles helped transform the texture of walnut meal products from rough and gritty into a fine, smooth powder, improving product quality while also expanding a wide range of application prospects.

A higher absolute zeta potential means a more stable dispersion system with stronger resistance to agglomeration; conversely, a lower absolute value makes the samples more prone to agglomeration [34]. Figure 2D shows the mean zeta potential of walnut meal after various ball milling times. Upon ball milling, the absolute values of the zeta potential for walnut meal were all lower than that of BM-0. This phenomenon differs from the effect of ball milling on isolated proteins, where the absolute zeta potential typically increases [30]. In the present study, the absolute zeta potential of walnut meal decreased after ball milling. This phenomenon is possibly attributed to the complex structure formed by insoluble dietary fiber and protein in walnut meal, which dominates its unique surface charge response. Fracture of the protein and fiber structures was caused by the ball milling process, and charged groups originally buried in the interior were exposed. The newly exposed groups carried a net charge opposite to that of the original particle surface; partial charge neutralization might occur, leading to a decrease in the measured zeta potential. Another possible mechanism was related to particle size. Smaller particles exhibit higher surface curvature. Under constant surface charge density, increased surface curvature might alter the structure of the electrical double layer and reduce the absolute zeta potential. A similar phenomenon has been reported in studies on wet-milled eggshells, where a decrease in particle size was accompanied by a decrease in the absolute zeta potential [35].

### 3.4. Microscopic Morphology and Structural Characteristics

#### 3.4.1. Microscopic Morphology

**Figure 3:**
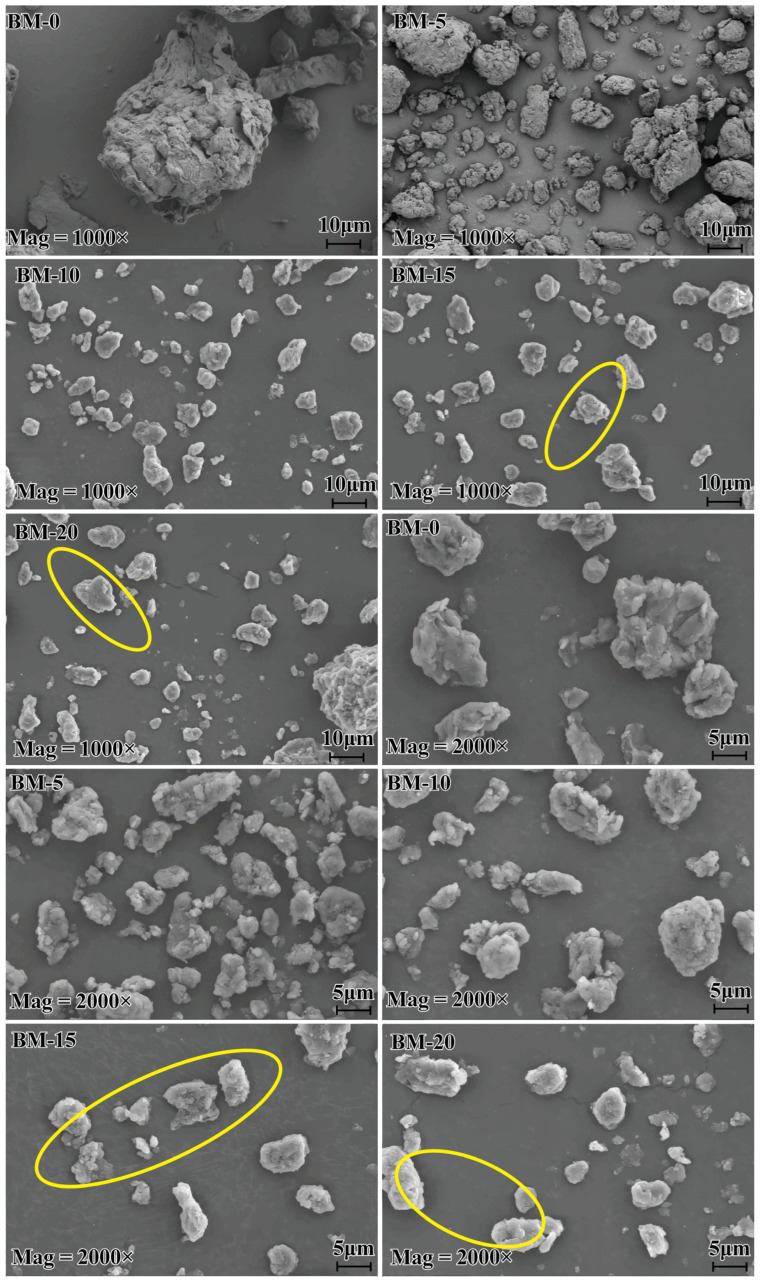
At magnifications of 1000× and 2000×, microstructural images of walnut meal after different ball milling times.

The microstructure of walnut meal changed with increasing ball milling time. It can be observed from Figure 3 that under the condition of 2000×, the particle size of BM-20 was larger than that of BM-15. This increase might be related to enhanced interparticle interactions, possibly involving surface form and functional groups [36]. At 1000×, BM-0 appeared relatively large, with intact protein and fiber structures, smooth surfaces, and few surface fissures. After ball milling, the walnut meal exhibited a fragmented morphology, rough surfaces, and a distinctly flaky appearance. Prolonged milling time led to the disappearance of the previously observed distinct fibrous strands. This change could be associated with the intimate mixing of fiber particles and proteins during milling, which diminished the apparent structural features of walnut meal [12,37].

#### 3.4.2. SDS-PAGE Analysis

**Figure 4:**
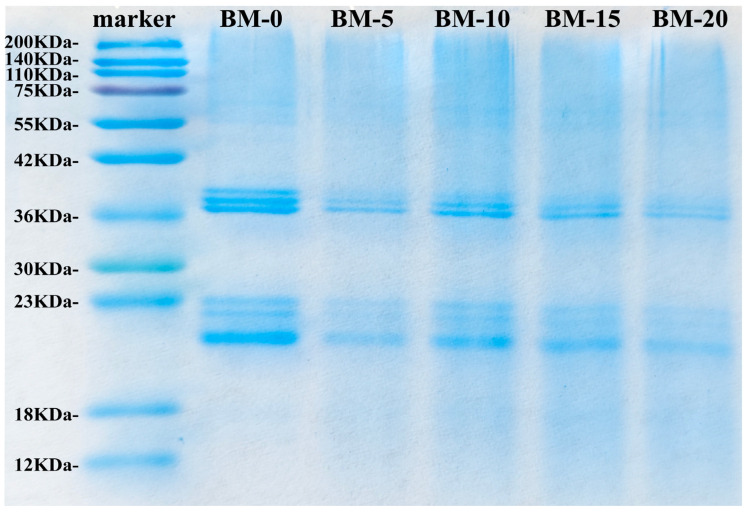
Changes in protein molecular weight in walnut meal subjected to different ball milling times.

Protein is one of the major components of walnut meal. Therefore, the protein profiles of samples subjected to different ball milling times were analyzed. The molecular weight distribution of proteins was mainly examined using SDS-PAGE. As shown in Figure 4, BM-0 mainly exhibited bands in the regions corresponding to glutenin subunits, including the alkaline subunit fraction (20–23 kDa) and the acidic glutenin subunit fraction (36–40 kDa). Clear bands were observed at both positions, while no distinct bands were detected above 42 kDa. After ball milling for different durations, no additional protein bands were observed, suggesting that ball milling, as a physical approach, does not hydrolyze peptide bonds and that the overall subunit composition remains essentially unchanged. However, slight decreases in the apparent molecular weights of some bands and variations in staining intensity were observed with increasing milling time. The decrease in apparent molecular weight observed with extended milling time might be attributed to altered subunit behavior, while the concurrent changes in electrophoretic patterns could reflect conformational rearrangements induced by mechanical forces, potentially reducing SDS binding and thus influencing gel migration. This treatment may have revealed exposed differences in disulfide bond-mediated aggregation or cross-linking among samples subjected to different ball milling times, contributing to the observed variations in band intensities and patterns [23,38].

#### 3.4.3. FTIR Analysis

Ball milling induced observable structural changes in walnut meal, with the degree of modification varying as a function of milling time. To investigate these conformational alterations, FTIR spectroscopy was employed to analyze the characteristic peaks of walnut meal subjected to 5, 10, 15, and 20 h of milling [39].

**Figure 5:**
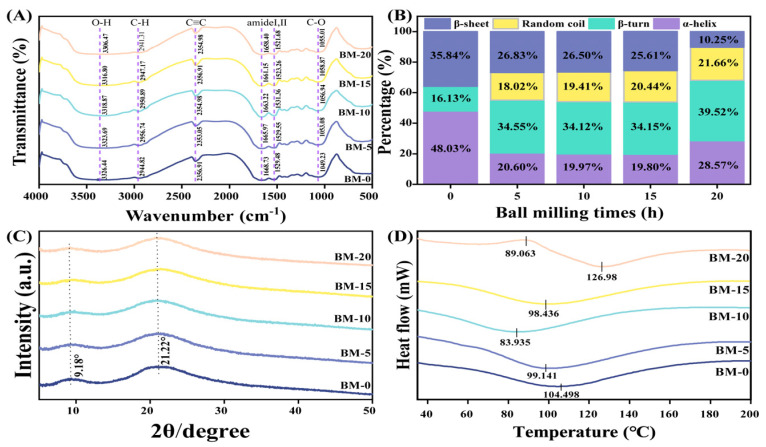
FTIR spectra (**A**), secondary structure of protein (**B**), XRD patterns (**C**), and DSC curves (**D**) of walnut meal prepared at different ball milling times.

As shown in Figure 5A, the BM-0 sample without ball milling treatment exhibited absorption peaks near 1050, 1538, 1650, 2350, 2900, and 3300 cm^−1^. The absorption band at approximately 1050 cm^−1^ corresponded to the asymmetric stretching of C–O, C–C, and C–O–C glycosidic bonds in cellulose and polysaccharides [40,41], while the peaks at 1650 cm^−1^ and 1538 cm^−1^ were assigned to the N–H bending and C–N stretching coupling in the amide II band region and the C=O stretching in the amide I band region, respectively [42]. The weak absorption band observed at approximately 2350 cm^−1^ was tentatively ascribed to the stretching vibration of C≡C or related multiple bonds. Given its low intensity and lack of supporting bands, this assignment should be considered preliminary. The peak near 2900 cm^−1^ was assigned to the C–H stretching vibration in proteins and IDF [43]. The broad band centered at approximately 3300 cm^−1^ originated from the overlapping stretching vibrations of O–H and N–H groups, which are characteristic of O–H and N–H functionalities in the sample [37].

Compared with BM-0, ball-milled walnut meal did not exhibit newly appeared absorption bands, indicating that no new major functional groups were introduced during the milling process. However, the absorption intensities of the characteristic peaks changed with milling time. As ball milling time increased, the absorption intensities of the protein amide I and amide II bands in walnut meal near 1650 cm^−1^ and 1538 cm^−1^ gradually decreased [44]. The weak absorption peak near 2350 cm^−1^ was consistent with C≡C groups or related multiple bonds. Since this peak was not inherent in the components of walnut meal, it might possibly have originated from heterocyclic or unsaturated compounds formed via the Maillard reaction induced by thermal energy during the high-temperature pressing process [45,46]. The absorption band near 3300 cm^−1^ decreased in intensity and shifted to lower wavenumbers (a redshift), which might be interpreted as a possible indication of stronger hydrogen bonding due to the overlap of O–H and N–H stretching vibrations with increasing ball milling time [47].

The percentages of protein secondary structures were obtained after analyzing the characteristic peaks of amide I and II. For the BM-0, no pronounced random coil structure was detected in the secondary structure profile. This observation may be attributable to the structural changes induced by the high-temperature pressing process, which might have resulted in increased proportions of α-helix and β-sheet content. When the ball milling time was prolonged to 15 h, the random coil content in the walnut meal powder increased to 20.44%. This observation suggests that ball milling could facilitate the unfolding of tightly associated secondary structures, which might be associated with inter-domain aggregation, as supported by the morphological changes observed in SEM, and thereby contribute to the increased random coil content both within and between protein molecules. Extending the ball milling time to 20 h further increased the contents of random coil and β-turn. These results suggest that ball milling treatment facilitates structural changes in the protein in walnut meal. These structural alterations could potentially contribute to the improvement of the functional properties of walnut meal.

#### 3.4.4. XRD Analysis

Figure 5C illustrates the XRD patterns of walnut meal obtained at different ball milling times. Diffraction peaks of BM-0 at 2θ = 20.293° and 8.963° were observed, corresponding to the 200 and 110 planes of crystalline cellulose [48]. The IDF associated with these two diffraction peaks can be classified as native cellulose [49]. After ball milling, the main diffraction peaks showed little change in position, while the secondary peaks exhibited noticeable broadening. Table 3 shows that the crystallinity corresponding to the main diffraction peak gradually increased from 6.09% to 6.75% as the ball milling time increased from 0 to 10 h, suggesting a slight upward trend. A decrease in crystallinity was observed in the BM-15 and BM-20, with values dropping to 5.22% and 4.22%, respectively. For the secondary diffraction peak, crystallinity fluctuated slightly between 2.29% and 2.56% from 0 to 15 h. However, for BM-20, the crystallinity of the secondary peak sharply declined to 0.74%. This sharp decline might be related to the disruption of interactions between cellulose and hemicellulose or lignin during ball milling, which could have promoted the transition of some crystalline cellulose into amorphous regions [50]. Overall, with increasing ball milling time, the crystallinity of walnut meal was relatively stable after short milling durations but decreased significantly with increasing milling time.

#### 3.4.5. Thermal Analysis

DSC is an effective technique for evaluating thermal stability [51]. As shown in Figure 5D, walnut meal of BM-0 to BM-15 exhibited endothermic transitions with peak temperatures at 104.498, 99.141, 83.915, and 98.436 °C, respectively. For BM-20, an exothermic peak at 89.063 °C and an additional endothermic peak at 126.98 °C were observed. For BM-0 to BM-10, the endothermic peak temperature gradually shifts toward the 80 °C range with increasing milling time. This shift might reflect alterations in the conformational stability of walnut meal components, potentially lowering the initial thermal transition temperature [52]. The high transition temperature observed for BM-15 might suggest enhanced thermal resilience of the protein matrix within walnut meal under intermediate milling conditions [53]. Notably, BM-20 exhibited an exothermic peak at 89.063 °C alongside an endothermic peak at 126.98 °C. The exothermic event could be associated with aggregation or molecular rearrangements of protein components induced by excessive mechanical stress, as opposed to a simple shift in denaturation temperature. The endothermic peak observed at 126.98 °C might be tentatively associated with alterations in the thermodynamic state of the IDF in walnut meal [54,55]. These thermal data indicate that ball milling substantially affected the thermal behavior of walnut meal components, with proteins and IDF displaying distinctly different response patterns during the milling process.

### 3.5. Physical and Chemical Properties and Functional Properties

#### 3.5.1. -SH Analysis

**Figure 6:**
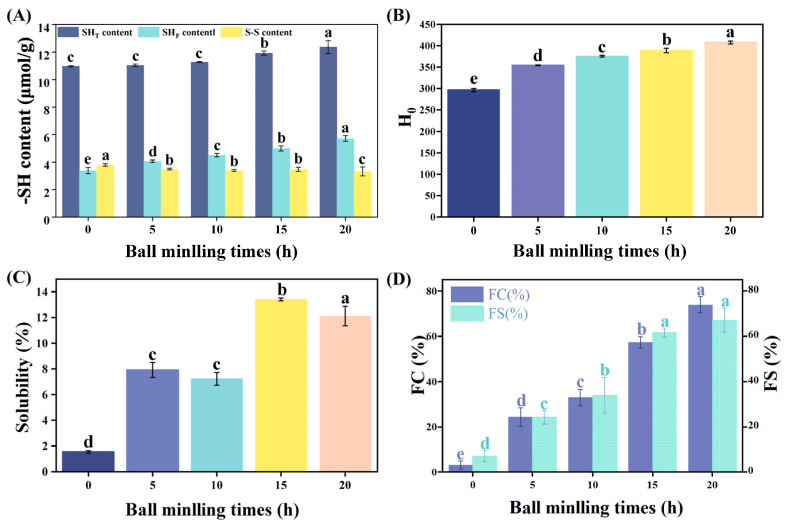
Content of -SH (**A**), H_0_ (**B**), solubility (**C**), and FC and FS (**D**) of walnut meal at different ball milling times. Different letters within each column indicate significant differences (*p* < 0.05) by Duncan’s test.

The -SH groups can engage in comparatively weak noncovalent interactions, and their level is one of the key indicators for protein unfolding and conformational stability [29]. As shown in Figure 6A, the -SH content in walnut meal continuously increased with prolonged ball milling time. The total -SH (-SH_T_) and free -SH (-SH_F_) contents in the BM-0 sample were 10.97 μmol/g and 3.38 μmol/g, respectively. After ball milling, the SH_T_ and SH_F_ contents increased. Compared with BM-0, the SH_T_ and SH_F_ contents in BM-20 increased to 12.02 μmol/g and 5.72 μmol/g, respectively. In contrast, the -S-S- content decreased from 3.79 μmol/g (BM-0) to 3.15 μmol/g (BM-20) after milling. These changes might have resulted from the fact that during the mechanical milling process, in addition to particle size reduction, the -S-S- bonds maintaining protein stability were disrupted, leading to a consequent increase in -SH content [56]. Moreover, prolonged ball milling may have simultaneously disrupted the rigid crystalline structure of IDF and exposed numerous -OH groups on its surface [57], a trend similar to that observed in the XRD results. Notably, the increase in -SH content was not linear; from BM-5 to BM-15, the increase was relatively slow, followed by a pronounced rise at BM-20. This change reflected that prolonged mechanical energy accumulation enabled a more complete dissociation of protein aggregates in walnut meal, with disulfide bond breakage extending from local to broader areas. Consequently, internal -SH groups were exposed in a concentrated manner [58]. These findings demonstrate that extended ball milling effectively altered the conformational state of proteins in walnut meal.

#### 3.5.2. H_0_ Analysis

H_0_ serves as a useful index for evaluating protein adsorption behavior at oil–water interfaces [59]. The H_0_ values for BM-0 to BM-20 were 300.85, 353.7, 375.30, 394.55, and 411.75, respectively, as illustrated in Figure 6B. Compared with BM-0, a steady and significant increase in H_0_ was noted with prolonged ball milling time. During the treatment from BM-0 to BM-5, the observed H_0_ value showed the largest increase. This might be because larger particle aggregates in walnut meal dissociated, the protein molecular structure expanded, and hydrophobic regions were exposed [60]. Subsequently, the increase in H_0_ from BM-10 to BM-20 became more gradual. It was speculated that the effect of ball milling transitioned from qualitative changes during the first 5 h to quantitative accumulation at longer durations, resulting in particle size refinement and internal structural disruption [61]. The excessive accumulation of mechanical forces during prolonged ball milling might have caused further alterations in protein structure. Apart from alterations in protein hydrophobic regions, another contributing factor could have been the presence of hydrophobic domains (e.g., lignin) in the IDF of walnut meal. Ball milling altered its crystalline structure; hydrophobic residues buried within the components may be fully exposed [57]. Additionally, as ball milling time increased, particle size decreased and specific surface area increased, providing more binding sites for fluorescent probes such as ANS and resulting in higher measured H_0_ values. The change in H_0_ trend was generally consistent with the changes in particle size and crystalline structure. As indicated by these results, ball milling significantly increased the H_0_ value of walnut meal, thereby improving its corresponding functional properties (e.g., foamability). Meanwhile, the increase in the H_0_ value of walnut meal also provides potential for its application as a bioactive carrier in functional foods.

#### 3.5.3. Solubility Analysis

As shown in Figure 6B, the solubility of BM-0 was only 1.54%, confirming the inherently low solubility of proteins and IDF. Extended ball milling time resulted in a pattern in which solubility first increased significantly and then slightly decreased. Starting from BM-5, solubility increased significantly to approximately 7%; it reached a peak of 13.04% at BM-15 and then decreased to 12.46% at BM-20 (*p* < 0.05). These results indicate that ball milling effectively improved the solubility, but excessive extension of ball milling time was not conducive to further enhancement of solubility.

The increase in solubility from BM-0 to BM-15 was possibly related to structural changes in both the protein and the insoluble dietary fiber within the walnut meal complex. The stable structure of the protein was affected by mechanical forces, becoming looser and unfolded, with an increase in polar regions on the protein surface. This was possibly associated with changes in the hydration capacity of walnut meal [62]. The decrease in solubility at BM-20 reflected the negative effects of over-milling. Excessive mechanical forces caused damage to the IDF structure, potentially accompanied by the exposure of more active groups (e.g., –OH groups). These exposed groups interacted with polar groups on the protein surface via hydrogen bonds or electrostatic interactions, and such interactions coincided with a reduction in the number of water-binding sites. On the other hand, secondary aggregation of particles led to the formation and precipitation of insoluble protein–fiber complexes, and this process was associated with the decrease in solubility [63]. The solubility results of walnut meal under different grinding times indicated that ball milling treatment could effectively improve the solubility of plant proteins. Studies (e.g., Sá et al. [64]) verified, using in vitro simulated digestion experiments, that protein solubility was positively correlated with digestibility. The increase in solubility facilitated protein digestion and absorption, thereby enhancing the utilization value of walnut meal as a functional ingredient.

#### 3.5.4. FC and FS Analysis

**Figure 7:**
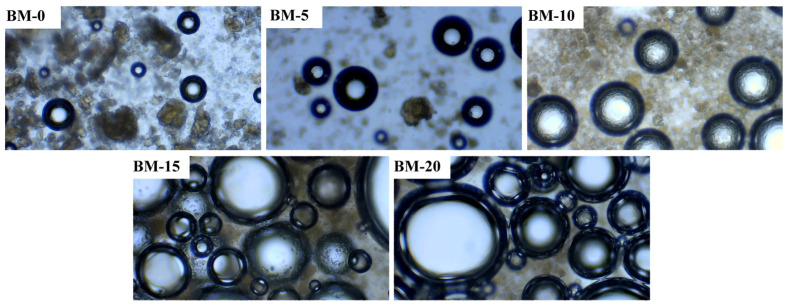
Schematic diagram of optical microscopy in walnut meal at different ball milling times.

FC is defined as the gas-trapping capacity of a suspension, while FS is the gas-retention capacity over time. The value of FC is governed by the film properties at the gas–liquid interface. As shown in Figure 6D, the FC of BM-0 was 3%, while those of BM-5 to BM-20 were 24.33%, 33.0%, 57.33%, and 74.0%, respectively. The FS of BM-0 was 8.33%, whereas those of BM-5 to BM-20 were 28.52%, 40.87%, 72.63%, and 78.99%, respectively (*p* < 0.05). With increasing ball milling time, the FC of walnut meal showed an overall increasing trend. Ball milling had a positive effect on improving the protein structure in walnut meal. Rapid stirring and an increase in H_0_ facilitated the rapid adsorption of proteins at the gas–liquid interface, leading to rapid conformational changes and rearrangements of the proteins [65]. The increase in ball milling time not only altered the protein structure in walnut meal but also promoted the conversion of IDF to SDF and increased the viscosity of the walnut meal solution [66]. Combined with the optical microscopy observations in Figure 7, it is speculated that the ultrafine particles provided an effective barrier between air bubbles, thereby reducing the rate of foam coalescence. Comparing BM-15 and BM-20, it was found that in the foam prepared from walnut meal milled for 15 h, IDF was more densely distributed, reducing contact between bubbles and more effectively reducing foam coalescence and rupture. In BM-20, due to factors such as particle agglomeration, IDF may have failed to form an effective foam barrier between bubbles, resulting in poorer FS. FC, FS, and optical microscopy results were obtained. Ball milling treatment improved the foaming properties of walnut meal under suspension conditions, yielding FC and FS values that are desirable for volume-expanding applications.

#### 3.5.5. Surface Tension Analysis

**Figure 8:**
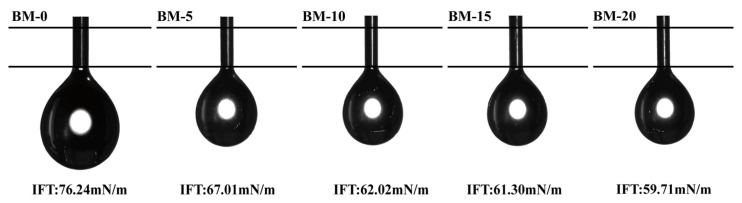
The schematic diagram of dynamic interfacial tension in walnut meal at different ball milling times.

Surface tension is an important parameter reflecting molecular arrangement and the magnitude of interaction forces at the gas–liquid interface [67]. As shown in Figure 8, the surface tension of BM-0 was 76.24 mN/m, while those of BM-5 to BM-20 were 67.01, 62.02, 61.30, and 59.71 mN/m, respectively. A decreasing trend was observed, indicating that the milling treatment exerted a synergistic modifying effect on walnut meal. The surface tension of a liquid is closely associated with the size and number of particles dispersed at the interface. One reason is that prolonged ball milling time reduces the particle size of its components while increasing the total specific surface area. This modifies the charge characteristics of the sample, thereby reducing electrostatic repulsion at the interface [68]. Another reason is that the structure of proteins in the walnut meal was disrupted, releasing surface-active substances. These substances migrate to the liquid phase and interact with dissolved oxygen in water to generate amphiphilic compounds, consequently lowering surface tension [69]. Due to the synergistic action of these two factors, the gas–liquid interface properties are modified, leading to a sustained reduction in the measured surface tension.

### 3.6. Pearson Correlation

**Figure 9:**
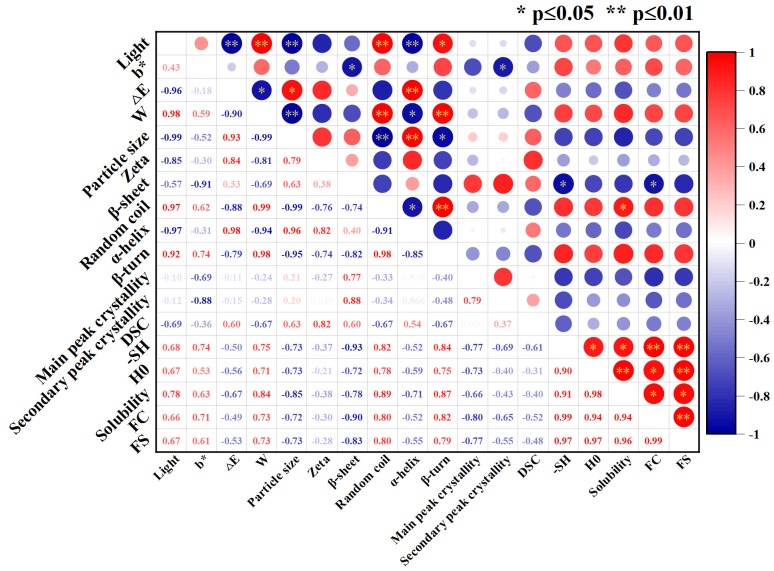
Pearson correlation matrix diagram of the structural and functional properties of walnut meal processed under different ball milling times.

Through correlation analysis, the relationship between the structural and functional properties of walnut meal was explored, with the strength of the Pearson correlation coefficients represented by color intensity. Given the limited number of milling time points (5, 10, 15, 20 h) evaluated in this study, the correlation analysis serves solely as an exploratory tool to examine the covariation trends between structural changes and functional properties. As shown in Figure 9, particle size and zeta potential showed negative correlations with -SH, H_0_, solubility, and foaming properties, while exhibiting positive correlations with color and brightness (*p* < 0.05). As a physical modification technique, ball milling effectively enhanced particle fragmentation and improved product color, but its direct contribution to changes in functional properties was relatively small. β-Sheet and α-helix showed negative correlations with -SH, H_0_, solubility, and foaming properties, whereas random coil and β-turn were positively correlated with these functional properties. Protein secondary structure is composed of β-sheet, α-helix, random coil, and β-turn. Ball milling induced changes in protein structure, increasing the proportions of β-turn and random coil. These changes were statistically correlated with indicators such as -SH, H_0_, solubility, and foaming properties. Ball milling further promoted structural changes in walnut meal proteins through high-speed rotational impact of the grinding media. In addition, crystallinity and DSC showed negative correlations with functional properties. Notably, ball milling treatment enhanced the intercorrelations among the functional properties (-SH, H_0_, solubility, FC, and FS), suggesting that these five parameters interact synergistically to improve the functional properties of walnut meal.

However, further validation using additional processing conditions (e.g., different ball milling parameters) is needed to better elucidate the relationship between structure and functional properties and to provide stronger references for the comprehensive utilization of walnut meal.

## 4. Conclusions

This study primarily investigated the effects of ball milling time on the structural and functional properties of walnut meal. As the ball milling time was prolonged, the brightness and whiteness of walnut meal were effectively improved. To determine the optimal ball milling time for walnut meal, multiple indicators from this study were taken as references, including achieving effective particle size reduction, moderate unfolding of protein structure, enhancement of functional properties, and avoidance of particle aggregation and performance deterioration caused by excessive milling. By comparing the data obtained at 15 h and 20 h of ball milling, it was found that extending the milling time from 15 h to 20 h led to increased particle size, decreased thermal stability, and deterioration in solubility and foam stability, although a few indicators continued to show marginal improvement. In contrast, walnut meal subjected to 15 h of ball milling achieved optimal performance across multiple parameters, exhibiting the smallest mean particle size and specific surface area, moderate protein structural unfolding coupled with maintained thermal stability, and increased levels of -SH content, H_0_, solubility, and foaming properties. Therefore, based on the performance optimization criteria, 15 h was determined to be the optimal ball milling time for walnut meal.

It is worth noting that excessive ball milling introduces industrial limitations such as reduced energy efficiency, increased equipment wear, and higher production costs. However, extending the operational window to approximately 20 h could offer practical advantages in industrial production by enabling more flexible scheduling and helping to mitigate the negative effects of raw material variability on product consistency. Therefore, under the premise of balancing product performance and processing feasibility, the ball milling time should be optimized according to the specific functional requirements of the final product so as to avoid unnecessary resource waste in industrial production.

## Figures and Tables

**Table 1:** Component contents of walnut meal (wet basis).

Component	Content (%)
Protein	68.85 ± 1.19
SDF	5.30 ± 0.95
IDF	11.77 ± 0.76
Moisture	4.89 ± 0.61
Ash	7.15 + 0.79

The data are expressed as the mean ± standard deviation.

**Table 2:** Color parameters and color differences in walnut meal at different ball milling times.

Sample	L⁎	a⁎	b⁎	∆E	W
BM-0	71.35 ± 0.29 ^d^	7.58 ± 0.19 ^a^	27.79 ± 0.21 ^d^	32.79 ± 0.38 ^a^	59.38 ± 0.39 ^e^
BM-5	83.50 ± 0.01 ^c^	6.24 ± 0.02 ^d^	28.84 ± 0.04 ^b^	28.82 ± 0.04 ^c^	66.20 ± 0.04 ^c^
BM-10	85.83 ± 0.13 ^a^	6.10 ± 0.06 ^d^	28.12 ± 0.11 ^c^	27.76 ± 0.13 ^d^	67.92 ± 0.16 ^a^
BM-15	84.82 ± 0.04 ^b^	6.45 ± 0.02 ^c^	28.24 ± 0.06 ^c^	28.10 ± 0.06 ^d^	67.30 ± 0.04 ^b^
BM-20	83.28 ± 0.18 ^c^	7.26 ± 0.05 ^b^	29.63 ± 0.09 ^a^	29.90 ± 0.13 ^b^	65.20 ± 0.17 ^d^

Walnut meal samples with different ball milling times were designated as BM-0, BM-5, BM-10, BM-15, and BM-20, respectively. The data are expressed as the mean ± standard deviation. Different superscript letters within the same column indicate significant differences (*p* < 0.05) by Duncan’s test.

**Table 3:** Crystallinity of walnut meal at different ball milling times.

Sample	Main Diffraction Peak Crystallinity (%)	Secondary Diffraction Peak Crystallinity (%)
BM-0	6.09 ± 0.04 ^c^	2.43 ± 0.08 ^b^
BM-5	6.30 ± 0.03 ^b^	2.29 ± 0.06 ^c^
BM-10	6.75 ± 0.02 ^a^	2.48 ± 0.04 ^b^
BM-15	5.22 ± 0.03 ^d^	2.562 ± 0.04 ^a^
BM-20	4.22 ± 0.02 ^e^	0.737 ± 0.05 ^d^

Results are presented as mean ± SD, and values in the same column with different superscripts differ significantly (*p* < 0.05) according to Duncan’s test.

## Data Availability

The original contributions presented in this study are included in the article/Supplementary Materials. Further inquiries can be directed to the corresponding author.

## Supplementary Materials

The following supporting information can be downloaded at: https://www.mdpi.com/article/10.3390/foods15132250/s1, Figure S1: At magnifications of 1000× and 2000×, microstructural images of walnut meal after different ball milling times. Figure S2: The schematic diagram of dynamic interfacial tension in walnut meal at different ball milling times.
