# Supplementary figures and images for: The Influence of the Ball Milling Process on the Structure and Functional Properties of Walnut Meal
Source: Foods. 2026 Jun 23;15(13):2250. doi: 10.3390/foods15132250 (PMC13361227; doi:10.3390/foods15132250)

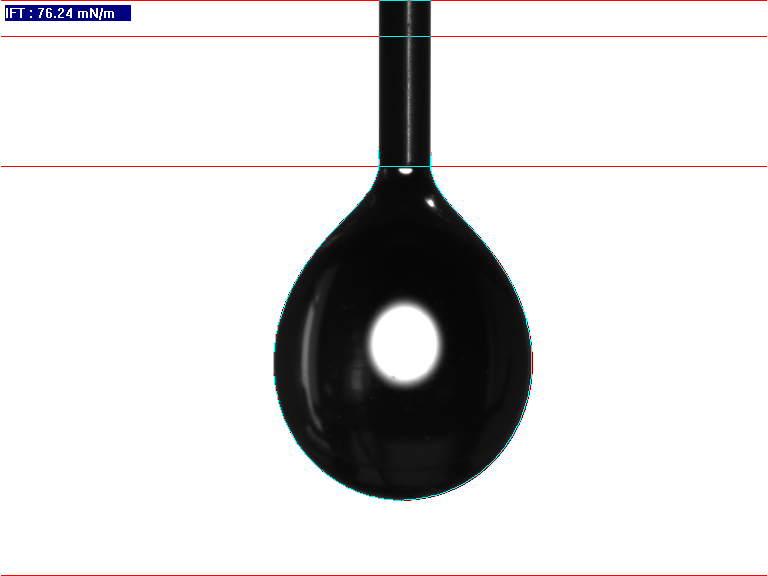

Supplement: Supplementary file 1 [file foods-15-02250-s001.zip › Figure S2. The schematic diagram of dynamic interfacial tension in walnut meal at different ball milling times/BM-0.bmp]

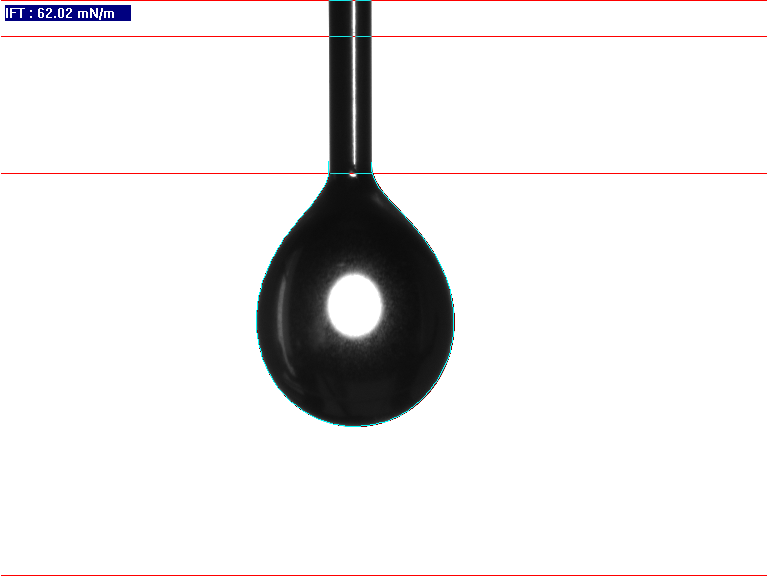

Supplement: Supplementary file 1 [file foods-15-02250-s001.zip › Figure S2. The schematic diagram of dynamic interfacial tension in walnut meal at different ball milling times/BM-10.bmp]

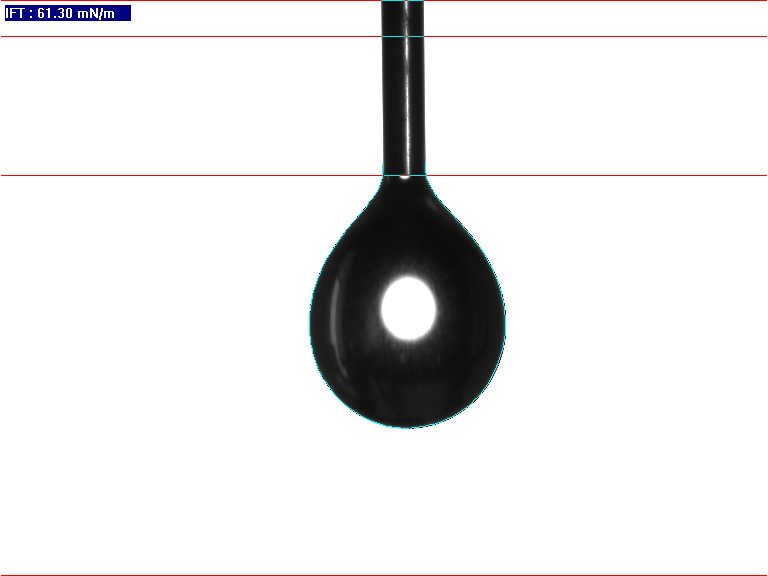

Supplement: Supplementary file 1 [file foods-15-02250-s001.zip › Figure S2. The schematic diagram of dynamic interfacial tension in walnut meal at different ball milling times/BM-15.bmp]

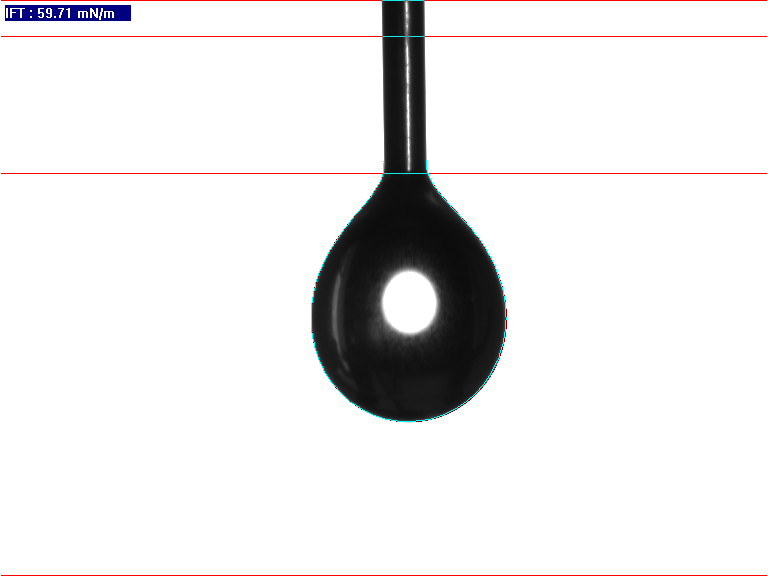

Supplement: Supplementary file 1 [file foods-15-02250-s001.zip › Figure S2. The schematic diagram of dynamic interfacial tension in walnut meal at different ball milling times/BM-20.bmp]

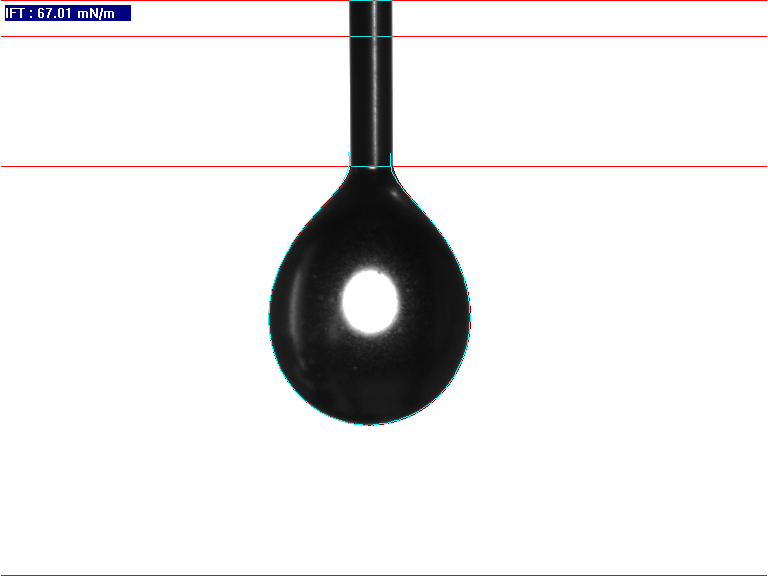

Supplement: Supplementary file 1 [file foods-15-02250-s001.zip › Figure S2. The schematic diagram of dynamic interfacial tension in walnut meal at different ball milling times/BM-5.bmp]
